# Hepatocyte growth factor mimetic protects lateral line hair cells from aminoglycoside exposure

**DOI:** 10.3389/fncel.2015.00003

**Published:** 2015-01-28

**Authors:** Phillip M. Uribe, Leen H. Kawas, Joseph W. Harding, Allison B. Coffin

**Affiliations:** ^1^Department of Integrative Physiology and Neuroscience, Washington State UniversityPullman, WA, USA; ^2^M3 Biotechnology, Inc.Seattle, WA, USA; ^3^College of Arts and Sciences, Washington State UniversityVancouver, WA, USA

**Keywords:** zebrafish, hair cell protection, hearing loss, aminoglycosides, hepatocyte growth factor, lateral line

## Abstract

Loss of sensory hair cells from exposure to certain licit drugs (e.g., aminoglycoside antibiotics, platinum-based chemotherapy agents) can result in permanent hearing loss. Here we ask if allosteric activation of the hepatocyte growth factor (HGF) cascade via Dihexa, a small molecule drug candidate, can protect hair cells from aminoglycoside toxicity. Unlike native HGF, Dihexa is chemically stable and blood-brain barrier permeable. As a synthetic HGF mimetic, it forms a functional ligand by dimerizing with endogenous HGF to activate the HGF receptor and downstream signaling cascades. To evaluate Dihexa as a potential hair cell protectant, we used the larval zebrafish lateral line, which possesses hair cells that are homologous to mammalian inner ear hair cells and show similar responses to toxins. A dose-response relationship for Dihexa protection was established using two ototoxins, neomycin and gentamicin. We found that a Dihexa concentration of 1 μM confers optimal protection from acute treatment with either ototoxin. Pretreatment with Dihexa does not affect the amount of fluorescently tagged gentamicin that enters hair cells, indicating that Dihexa’s protection is likely mediated by intracellular events and not by inhibiting aminoglycoside entry. Dihexa-mediated protection is attenuated by co-treatment with the HGF antagonist 6-AH, further evidence that HGF activation is a component of the observed protection. Additionally, Dihexa’s robust protection is partially attenuated by co-treatment with inhibitors of the downstream HGF targets Akt, TOR and MEK. Addition of an amino group to the N-terminal of Dihexa also attenuates the protective response, suggesting that even small substitutions greatly alter the specificity of Dihexa for its target. Our data suggest that Dihexa confers protection of hair cells through an HGF-mediated mechanism and that Dihexa holds clinical potential for mitigating chemical ototoxicity.

## Introduction

Hearing loss and vestibular dysfunction are some of the most prevalent and debilitating disorders due to social isolation resulting from sensory deprivation. Particularly among the elderly, those with untreated hearing loss are less likely to participate in social activities and more likely to report levels of depression and anxiety (Bess et al., 1989; Knutson and Lansing, 1990). Hearing loss can result from excessive exposure to loud noise, genetic factors, or exposure to certain licit drugs, termed ototoxins, such as aminoglycoside antibiotics and platinum-based chemotherapeutics like cisplatin (Fee, 1980; Rybak et al., 2009). Due to their high efficacy, many ototoxins are still used despite the risk of hearing loss. Aminoglycoside antibiotics are still widely used for a variety of clinical applications. For example, cystic fibrosis patients suffer from severe gram-negative bacterial infections and are commonly prescribed the aminoglycoside antibiotics amikacin or tobramycin, despite as high as a 14% incidence of hearing loss (Cheng et al., 2009). Cisplatin use carries an even higher reported incidence of hearing loss, between 28–68%, but it is used to treat several types of soft tissue tumors and is the most commonly prescribed chemotherapeutic for ovarian cancer (Rybak, 2007; Musial-Bright et al., 2011).

The primary cellular basis for ototoxic hearing loss is the death of sensory hair cells within the cochlea. Hair cells are mechanosensitive receptors in the inner ear that detect sounds and head movements; in mammals, loss of these cells is permanent and irreversible. There are currently no food and drug administration (FDA) approved treatments for the prevention of toxin-induced hair cell loss. Identification of a preventative therapy would allow for continued use of these efficacious antibiotics and chemotherapeutics without the devastating ototoxic consequences that accompany their use. Our work identifies a new otoprotective drug that is already designed for the clinical environment, allowing for quick transition to clinical use.

Studies involving acoustic and chemical insults show that growth factors represent a compelling class of otoprotective compounds (Yagi et al., 1999; Shoji et al., 2000; Kawamoto et al., 2003; Liu et al., 2008). Hepatocyte growth factor (HGF), a potent neurotrophic factor, affects a variety of neuronal cell types to promote axon guidance and cell survival (Ebens et al., 1996; Miyazawa et al., 1998). Direct application of exogenous HGF onto cochlear explants protects hair cells from neomycin ototoxicity (Kikkawa et al., 2009). However, HGF itself does not represent a viable preventative therapeutic due to low blood-brain barrier permeability and a half-life of only 3.8 min (Appasamy et al., 1993). One study attempted to circumvent these constraints by using viral HGF gene delivery to the inner ear to drive constitutive HGF expression (Oshima et al., 2004). This viral HGF gene delivery approach protected rat cochlear hair cells from kanamycin ototoxicity, further evidence for a protective role of HGF in the auditory periphery. Unfortunately, viral gene therapy presents the risk of insertional mutagenesis and does not reliably yield high expression profiles (Monahan and Samulski, 2000). However, development of a small molecule capable of stimulating HGF, and its associated receptor c-Met, may be clinically efficacious.

The HGF mimetic Dihexa is a synthetically derived Angiotensin IV analog that is blood-brain barrier permeable, stable, and orally bioavailable (McCoy et al., 2013). McCoy et al. (2013) found that Dihexa treatment ameliorated memory loss in a rat model of Parkinson’s Disease. Dihexa can bind to and dimerize HGF, forming a functional ligand and thereby activating c-Met (Benoist et al., 2014). HGF signaling is linked to neural protection in rodents both *in vitro* and *in vivo*, making a strong case for a small molecule activator of HGF/c-Met to confer a neuroprotective benefit (Zhang et al., 2000; Niimura et al., 2006). Here, we examine the otoprotective effects of Dihexa in a zebrafish model of chemical ototoxicity.

The experimental value of zebrafish as an animal model for *in vivo* drug discovery has grown rapidly due to their ease of assessment, large clutch size, and ability to recapitulate human disease conditions (MacRae and Peterson, 2003). The larval zebrafish lateral line is an especially tractable model for the identification of compounds that prevent hair cell loss (Coffin et al., 2010; Esterberg et al., 2013). The lateral line system of teleost fishes is an externally located, mechanosensitive sensory system used to detect vibrations in the aquatic environment that aid in predator detection, prey avoidance, and schooling behavior (Coombs et al., 1989). Hair cells of the lateral line are structurally and functionally similar to those of the mammalian inner ear and, perhaps more importantly, respond similarly to toxins (Harris et al., 2003; Ou et al., 2007; Coffin et al., 2013b). Previous drug discovery work in the larval zebrafish lateral line identified the novel compound PROTO-1 that prevents aminoglycoside-induced hair cell toxicity in rodent models *in vivo*, demonstrating that otoprotective drug discovery in zebrafish translates to mammalian systems (Owens et al., 2008; Rubel et al., 2011).

In this study, we demonstrate that Dihexa protects lateral line hair cells from aminoglycoside ototoxicity. Dihexa does not alter the entry of aminoglycosides into hair cells but rather attenuates cell death through an HGF-dependent signaling mechanism. This work demonstrates the potential clinical utility of Dihexa as a co-administered protectant to prevent aminoglycoside ototoxicity.

## Materials and methods

### Animals

Larval zebrafish (*AB) were obtained through pair-wise matings and raised at 28.5°C in petri dishes (Westerfield, 2000). All zebrafish were maintained on a 14 h light/10 h dark cycle in the Coffin Lab zebrafish facility at Washington State University, Vancouver. Embryos were raised until 5 or 6 days post-fertilization (dpf) prior to experimentation because larvae younger than 5 dpf exhibit some resistance to aminoglycoside ototoxicity (Murakami et al., 2003; Santos et al., 2006). Transgenic Brn3c:mGFP zebrafish were used for direct hair cell counts and c-Met localization studies. Brn3c:mGFP fish express membrane bound GFP in hair cells under control of the Brn3c (aka Pou4f3) promoter (Xiao et al., 2005). Previous work has demonstrated that GFP positive cells in Brn3c:mGFP fish co-label with phalloidin staining and thus can be used a reliable marker of mature hair cells (Uribe et al., 2013). All experimental procedures were approved by the Washington State University Animal Care and Use Committee.

### HGF receptor localization

Immunohistochemistry was performed using 1:500 anti-Met (Cell Signaling, Danvers, MA). Following fixation in 4% paraformaldehyde (PFA), 5 dpf Brn3c:mGFP larvae, which express membrane-bound GFP in hair cells, were rinsed twice with phosphate-buffered saline (PBS) for 10 min each and then once with dH_2_o for 20 min. Larvae were then placed into blocking solution, which consisted of 5% goat serum in PBST (0.1% Triton x-100, Sigma) for 1 h. Fish were then incubated overnight at 4°C in anti-Met in PBST with 1% goat serum. Excess primary antibody was washed off by three 10 min PBST rinses. Fish were then incubated for 4 h in 1:500 Alexa Fluor 568 secondary antibody (Life Technologies) diluted in PBST at room temperature. Excess secondary antibody was washed off by 3 consecutive 10-min rinses with PBST. Labeled larvae were then rinsed once with PBS and stored in 1:1 PBS:glycerol for confocal imaging. Adult *AB liver was removed and fixed for identical tissue processing (Gupta and Mullins, 2010).

### Drug treatments

Neomycin (10 mg/mL) and gentamicin (50 mg/mL) solutions (Sigma-Aldrich; St. Louis, MO) were diluted in E2 embryo medium (EM; 1 mM MgSO_4_, 120 μM KH_2_PO_4_, 74 μM Na_2_HPO_4_, 1 mM CaCl_2_, 500 μM KCl, 15 mM NaCl, and 500 μM NaHCO_3_ in dH_2_O; Westerfield, 2000) to working concentrations of 25–400 μM. Cisplatin stock (1 mg/mL; WG Critical Care; Paramus, NJ) was diluted in EM to working concentrations of 500–1000 μM and the pH adjusted to 7.2, since the cisplatin stock is highly acidic. Dihexa was synthesized by RS Synthesis (Louisville, KY) with a purity of 95.1%.

Free swimming zebrafish larvae were placed in 6-well plates and exposed to ototoxin in the presence or absence of Dihexa. Fish were pretreated with Dihexa or vehicle dimethyl sulfoxide (DMSO) control for 1 h. Fish were then co-treated with Dihexa or DMSO and neomycin (30 min), acute gentamicin (30 min), chronic gentamicin (6 h), or cisplatin (4 h). Ototoxin exposure times were selected based on previous work (Ou et al., 2007; Coffin et al., 2009; Owens et al., 2009). Fish were then rinsed three times in EM. Gentamicin (chronic) and cisplatin exposed groups and associated controls were assessed immediately following the three EM washes. Fish treated acutely with neomycin or gentamicin, and their associated controls, were allowed to recover in EM for 45 min prior to hair cell assessment.

### Hair cell assessment

Survival of lateral line hair cells was assessed by vital dye labeling in live fish. The vital dye 2-(4-(dimethylamino)styryl)-*N*-ethylpyridinium iodide (DASPEI) (Life Technologies, Grand Island, NY) is a marker of mitochondrial membrane potential and preferentially stains lateral line hair cells when added to the surrounding EM (Harris et al., 2003). Fish were incubated in 0.005% DASPEI for 15 min, then rinsed twice with EM and anesthetized with 0.001% MS-222 (Argent Labs, Redmond, WA). Using a Leica M165FC fluorescence dissection scope, 10 anterior neuromasts (IO1, IO2, IO3, IO4, M2, MI1, MI2, O2, SO1, and SO2; see Raible and Kruse, 2000) per fish were assessed based on fluorescent intensity (Harris et al., 2003; Coffin et al., 2009; Owens et al., 2009). An intensity score of 2 signifies intense neuromast fluorescence, an intermediate score of 1 represents faint DASPEI labeling, while a 0 neuromast score equates to the absence of neuromast fluorescence at a given neuromast’s stereotyped position. The scores from 10 neuromasts for each fish were summed such that each larva receives a final score between 0 (no neuromast fluorescence) and 20 (full complement of hair cells in all 10 neuromasts). To obtain direct hair cell counts from Brn3c:mGFP fish, we fixed larvae post-treatment in 4% PFA overnight at 4°C. Fish were rinsed with PBS three times, placed in 1:1 PBS:glycerol, and mounted on bridged coverslips. Hair cell counts for 5 anterior neuromasts (IO1, IO2, IO3, M2, and OP1) per fish were obtained and summed.

### Aminoglycoside uptake

Gentamicin conjugated to the fluorophore Texas Red (GTTR) was used to determine if Dihexa affected aminoglycoside uptake by hair cells (Steyger et al., 2003). Larvae were pretreated with Dihexa or DMSO for 1 h, then co-treated with GTTR for 3 min, rinsed 4 times in EM for 30 s each, and fixed in 4% PFA overnight at 4°C (Owens et al., 2008). GTTR-labeled neuromasts were visualized using a Leica SP8 confocal microscope, keeping gain and laser intensity values constant for a given experiment. Z-stack images of O2, MI1, and MI2 neuromasts were collected and compressed using Leica LAS AF software. Neuromasts were masked using a line tool and mean fluorescence was measured for an entire neuromast using ImageJ version 1.48 (National Institutes of Health, Bethesda, MD), mean background fluorescence was then subtracted out to yield net mean fluorescence for each neuromast.

### Pharmacological inhibitors

Pharmacological inhibition of HGF/c-Met or its downstream targets was used to elucidate which factors are important for Dihexa-mediated otoprotection. All inhibitors used were co-administered with Dihexa, that is 1 h pretreatment and co-treatment with a given ototoxin. 6-AH is a metabolically stable antagonist to the HGF/c-Met system that works by preventing HGF dimerization and activation (Kawas et al., 2012). We used 1 μM 6-AH as this concentration did not affect fish health or alter hair cell loss in response to neomycin but did attenuate neomycin-induced hair cell death. We used 10 μM rapamycin, a TOR inhibitor, (GenDEPOT, Barker, TX) as this concentration had no affect on neomycin ototoxicity or fish health but attenuated Dihexa hair cell protection in preliminary experiments. Rapamycin concentrations as high as 50 μM were tested initially and no overall toxicity was observed. We conducted similar optimization experiments with the MEK inhibitor, UO126 and an Akt inhibitor (CAS 612847-09-3; both inhibitors from EMD Millipore, Billerica, MA), selecting 1 μM as the optimal concentration of each compound. For both UO126 and Akt inhibitor, the highest concentration tested was 10 μM and we observed no overt toxicity at this concentration.

### Statistical analysis

Data were analyzed using GraphPad Prism (V. 6.0, La Jolla, CA). Statistical analyses were performed using either an un-paired *t*-test assuming equal variance, one-, or two-way ANOVA, as appropriate, and are specifically indicated on each figure legend. *Post hoc* comparisons were performed using Bonferonni corrections. Statistical values for ototoxin were omitted from figure legends because it is established that they cause a dose-dependent decrease in hair cell survival, in other words, we know that a dose-response curve for neomycin only will be statistically significant. All data are presented as mean ± s.e.m.

## Results

### c-Met expression in larval zebrafish neuromasts

**Figure 1:**
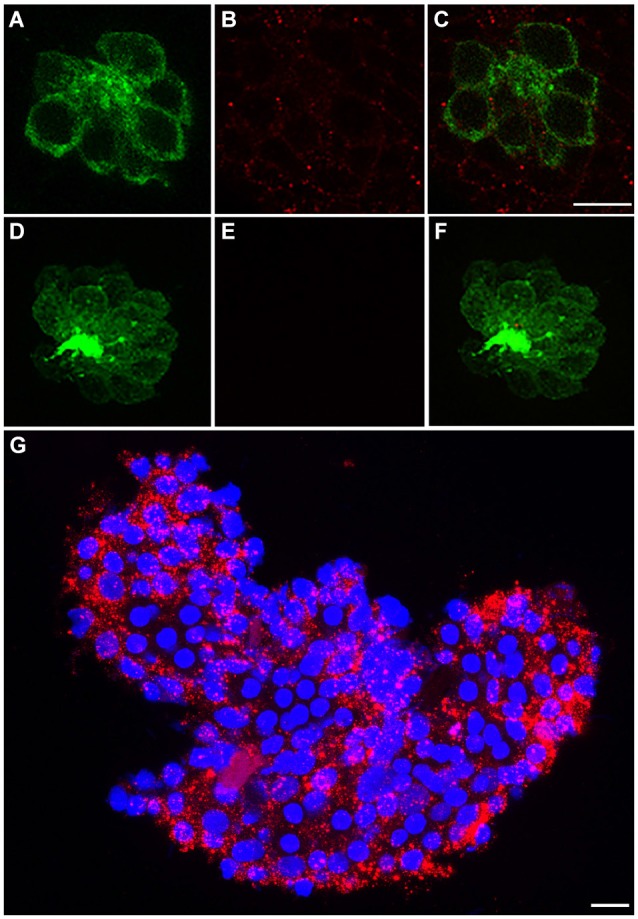
**c-Met is expressed in lateral line neuromasts. (A)** Neuromast of a Brn3c:mGFP transgenic zebrafish with clearly labeled hair cell boundaries. **(B)** Anti-c-Met labeling (red punctae) is present throughout the neuromast. **(C)** Merged image shows c-Met is present near the hair cell membrane and in surrounding cells. **(D–F)** Brn3c:mGFP larvae incubated with secondary antibody only show no c-Met labeling. **(G)** *AB adult liver tissue labeled with DAPI (blue) and anti-c-Met (red) demonstrates robust, punctate c-Met expression. Scale bar in **(C)** represents 5 μm and applies to images **(A–F)**. Scale bar in **(G)** represents 5 μm.

We immunohistochemically processed 5 dpf Brn3c:mGFP larvae using anti-c-Met to determine if c-Met expression is present in anterior lateral line neuromasts. In these fish, GFP localizes to the hair cell membrane, allowing for easy delineation of cell boundaries (Figure 1A). c-Met expression was widespread in the skin epithelium, including in neuromasts (red punctae in Figure 1B). No red punctae were present in a secondary antibody only control that was not incubated in anti-c-Met (Figures 1D–F). c-Met labeling is present both in hair cells and in other cell types in the neuromast (Figure 1C). To verify the validity of observed c-Met expression in hair cells, adult *AB liver tissue was immunohistochemically processed for anti-c-Met and DAPI (blue). c-Met expression in the adult liver is widespread and punctate, similar to the neuromast expression profile (Figure 1G). These results indicate that our c-Met labeling is reliable and that anterior lateral line neuromasts should be receptive to c-Met modulation.

### Dihexa protects lateral line hair cells from acute aminoglycoside toxicity

**Figure 2:**
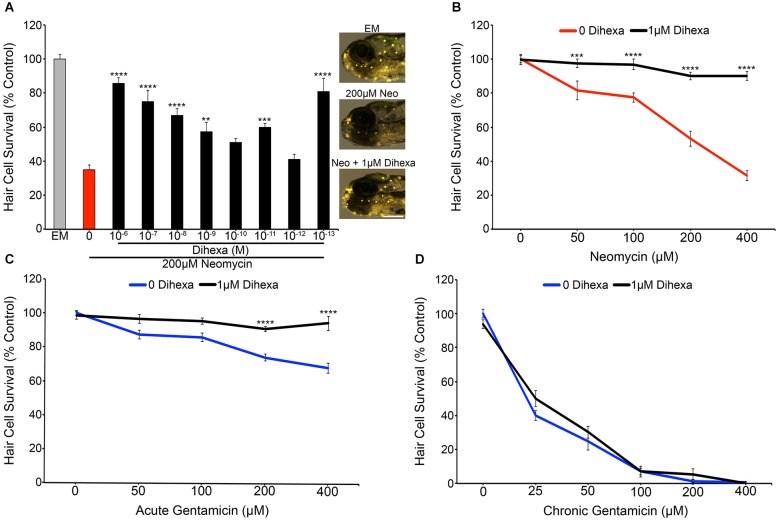
**Dihexa protects hair cells from acute aminoglycoside treatment. (A)** Dihexa confers dose-dependent protection from 200 μM neomycin with two peaks of protection at 10^−6^ M (1 μM) and 10^−13^ M (100 fM), with six concentrations of Dihexa providing significant protection (One-way ANOVA; *F*_(9,70)_ = 20.53 *p* < 0.001). Control fish exposed to embryo medium (EM) only (inset top) and 1 μM Dihexa plus 200 μM neomycin (inset bottom) display bright DASPEI fluorescence while fish treated with 200 μM neomycin alone (inset middle) display dim to absent DASPEI fluorescence. Scale bar represents 250 μm and applies to all three images in **(A). (B)** 1 μM Dihexa provides robust protection from aminoglycoside treatment across multiple concentrations of neomycin (Two-way ANOVA; Dihexa: *F*_(1,63)_ = 155.8 *p* < 0.001). **(C)** 1 μM Dihexa confers significant protection from acute gentamicin exposure across all concentrations of gentamicin tested (Two-way ANOVA; Dihexa: *F*_(1,71)_ = 58.42 *p* < 0.001). **(D)** 1 μM Dihexa does not provide protection against chronic gentamicin exposure (Two-way ANOVA; Dihexa: *F*_(1,81)_ = 1.458 *p* > 0.05). Asterisks indicate significant difference from aminoglycoside only control (*p < 0.05, **p < 0.01, ***p < 0.005, ****p < 0.001). *N* = 7–11 animals per treatment, error bars represent ± s.e.m.

Treatment with Dihexa confers protection from neomycin in a dose-dependent manner (Figure 2A). Untreated controls labeled with the vital dye DASPEI display bright neuromast fluorescence (Figure 2A inset). In contrast, animals treated with 200 μM neomycin for thirty minutes exhibit dim or completely absent fluorescence. The greatest protection was found at 10^−6^ M (1 μM), where only a minor decrease from control hair cell survival scores was observed. An additional peak of protection was seen at 10^−13^ M (100 fM) but was inconsistent across experiments, so all additional experiments were run with 10^−6^ M (1 μM) Dihexa. Figure 2B shows that 10^−6^ M (1 μM) Dihexa significantly protects hair cells from a range of neomycin concentrations. Additionally, there was no observed toxicity of Dihexa treatment alone. Dihexa also protects hair cells from variable concentrations of acute gentamicin (Figure 2C).

Previous work has demonstrated that aminoglycosides activate acute and slow (chronic) mechanisms that are distinct from one another, with neomycin only activating acute mechanisms but gentamicin activating both pathways (Coffin et al., 2009, 2013b; Owens et al., 2009). To ask if Dihexa can modulate both mechanisms of aminoglycoside-induced hair cell death, Dihexa pretreatment was followed by 6 h chronic gentamicin co-treatment. Dihexa was not protective against chronic gentamicin exposure (Figure 2D). Since slow-acting cell death processes can proceed following ototoxin removal (Owens et al., 2009), we next asked if Dihexa-mediated protection was still present 6 h after neomycin exposure. Dihexa-mediated protection was still robust 6 h post-neomycin washout (data not shown).

**Figure 3:**
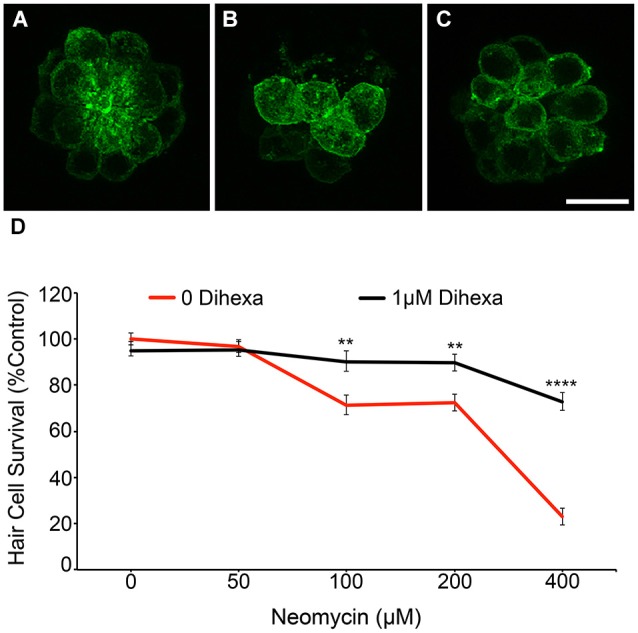
**Dihexa treatment prevents neomycin-induced hair cell loss in Brn3c:mGFP transgenic larvae. (A)** DMSO-treated control, **(B)** 400 μM neomycin treated neuromast shows a reduction in hair cells, and **(C)** neuromasts treated with 1 μM Dihexa plus 400 μM neomycin remain mostly intact. **(D)** 1 μM Dihexa provides protection from neomycin-induced hair cell loss across the neomycin dose-response curve (Two-way ANOVA; Dihexa: *F*_(1,456)_ = 50.77 *p* < 0.001). Asterisks indicate significant difference from neomycin control (**p < 0.01, ****p < 0.001). *N* = 8–10 animals per treatment, error bars represent ± s.e.m.

To verify DASPEI scores, fish from the same treatment groups were fixed and immunohistochemically processed with anti-parvalbumin (Millipore) to visualize hair cells (see Coffin et al., 2013b for details) (data not shown). Direct hair cell counts were also obtained from Brn3c:mGFP fish, further demonstrating that Dihexa robustly protects hair cells from neomycin damage (Figure 3).These results indicate that Dihexa only modulates targets responsible for the acute phase of aminoglycoside ototoxicity.

### Dihexa does not block gentamicin uptake into hair cells

**Figure 4:**
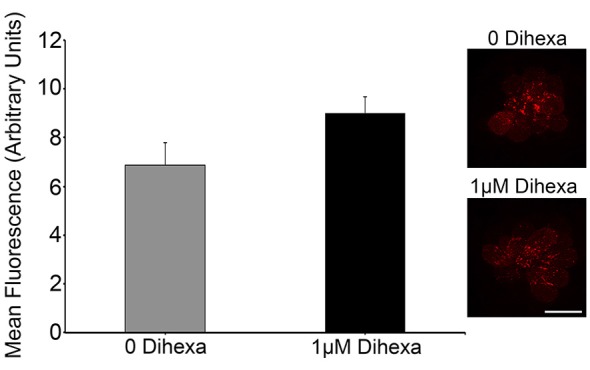
**Dihexa does not block aminoglycoside entry into hair cells**. There was no significant difference in quantified GTTR fluorescence within neuromasts of animals treated with or without 1 μM Dihexa (*t*-test; *p* = 0.1158). Sample control neuromast with bright GTTR fluorescence (inset top) is qualitatively the same intensity as a neuromast treated with 1 μM Dihexa (inset bottom). Scale bar represents 10 μm and applies to both images. *N* = 8 animals per treatment, error bars represent ± s.e.m.

Certain otoprotectants, namely those with quinolone rings, have been observed to confer protection by blocking aminoglycoside entry into hair cells instead of modulating cell death targets (Ou et al., 2009, 2012). To test if Dihexa blocks aminoglycoside uptake by hair cells, we quantified GTTR fluorescence either with or without 1 μM Dihexa. GTTR uptake was not altered in the presence of Dihexa, as seen in the qualitative examples and quantitative fluorescence data shown in Figure 4. This result indicates that Dihexa likely protects hair cells by modulating a cell death target.

### Dihexa otoprotection is mediated by HGF/c-Met signaling

**Figure 5:**
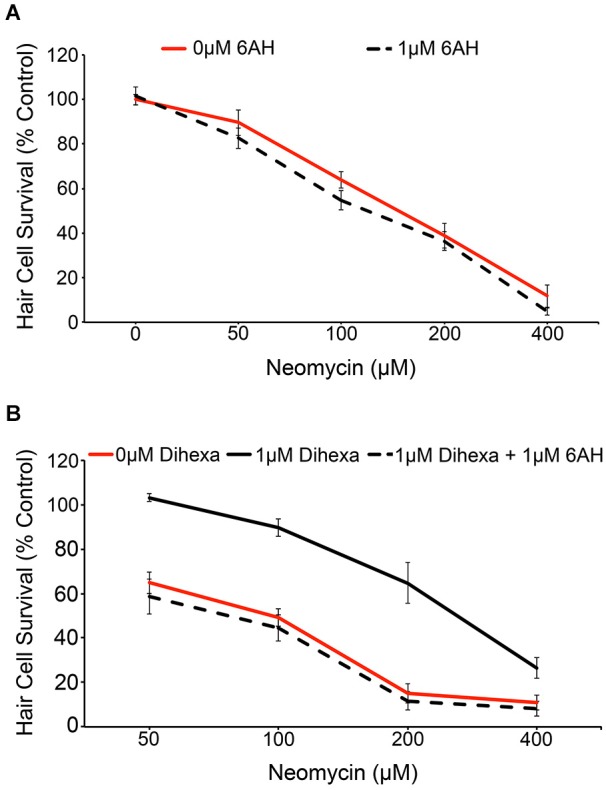
**Dihexa-mediated protection is inhibited by the HGF antagonist 6-AH. (A)** Treatment with 1 μM 6-AH does not alter lateral line hair cell survival in response to neomycin (Two-way ANOVA; 6-AH: *F*_(1,72)_ = 3.255 *p* = 0.0754). **(B)** Treatment with 1 μM Dihexa decreases neomycin-induced hair cell death over multiple concentrations of neomycin. Co-treatment with 6-AH completely attenuates Dihexa-mediated protection (Two-way ANOVA; Dihexa: *F*_(2,76)_ = 76.30 *p* < 0.001). There is a significant difference (*p* < 0.001) when comparing 1 μM Dihexa vs. 1 μM Dihexa plus 1 μM 6-AH at 50, 100, and 200 μM neomycin treatment groups, *p* < 0.05 at 400 μM neomycin. *N* = 6–9 animals per treatment, error bars represent ± s.e.m.

We used the pharmacological inhibitor 6-AH to investigate if the observed protection conferred by Dihexa is actually mediated by HGF/c-Met signaling. 6-AH blocks HGF dimerization and thereby inhibits subsequent c-Met activation (Kawas et al., 2012). Treatment with 6-AH alone does not alter hair cell survival in response to neomycin (Figure 5A). Co-treatment of larvae with 6-AH and Dihexa completely attenuates the hair cell protection conferred by Dihexa treatment (Figure 5B). This observation implicates the requirement of an active HGF/c-Met signal in Dihexa otoprotection.

### Downstream cellular mediators of Dihexa protection

**Figure 6:**
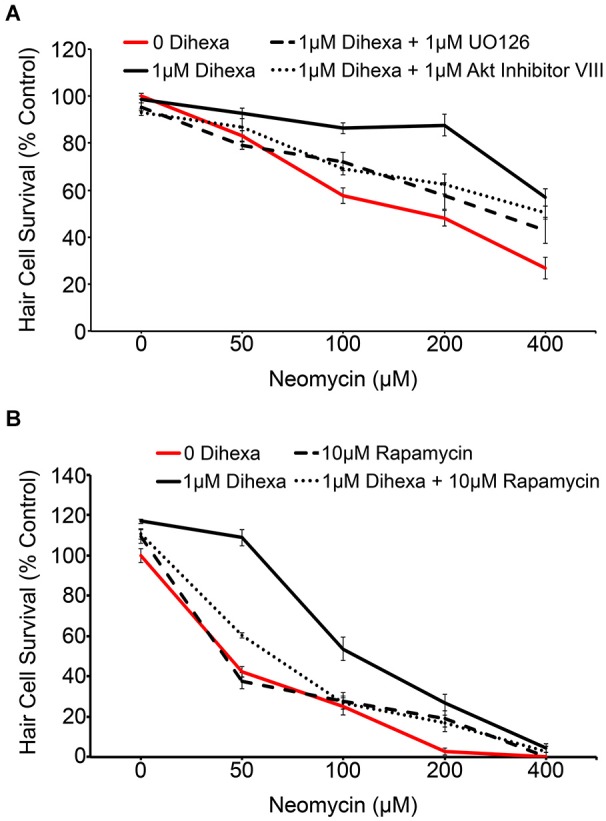
**Dihexa-mediated protection is inhibited by MEK, Akt, and TOR inhibitors. (A)** Co-treatment with 1 μM MEK or Akt inhibitors (UO126 and Akt Inhibitor VIII, respectively) partially attenuates Dihexa-dependent protection (Two-way ANOVA; Dihexa: *F*_(3,144)_ = 37.13 *p* < 0.001). When comparing the 1 μM Dihexa treatment vs. 1 μM Dihexa plus 1 μM UO126 *p*-values are as follows: 50 μM neomycin (*p* < 0.05), 100 μM neomycin (*p* < 0.05), 200 μM neomycin (*p* < 0.001), and 400 μM neomycin (*p* < 0.05). When comparing the 1 μM Dihexa treatment vs. 1 μM Dihexa plus 1 μM Akt inhibitor VIII *p*-values are as follows: 50 μM neomycin (*p* > 0.05), 100 μM neomycin (*p* < 0.05), 200 μM neomycin (*p* < 0.001), 400 μM neomycin (*p* > 0.05). **(B)** Co-treatment with 10 μM of the TOR inhibitor, rapamycin, also partially attenuates Dihexa-mediated protection from neomycin (Two-way ANOVA; Dihexa: *F*_(3,120)_ = 70.41 *p* < 0.001). When comparing the 1 μM Dihexa treatment vs. 1 μM Dihexa plus 10 μM rapamycin *p*-values were as follows: 50 μM neomycin (*p* < 0.001), 100 μM neomycin (*p* < 0.001), 200 and 400 μM neomycin (*p* > 0.05). *N* = 6–9 animals per treatment, error bars represent ± s.e.m.

Activation of the receptor tyrosine kinase c-Met leads to the recruitment of many signaling mediators and ultimately the activation of downstream signals, most notably Akt-TOR and Ras-ERK (Organ and Tsao, 2011). The MAPK and Akt inhibitors, UO126 and Akt inhibitor VIII respectively, were used to determine to what extent, if any, activation of their targets is required for Dihexa-mediated protection. Optimal concentrations of UO126 and Akt Inhibitor VIII (1 μM) were determined based on the highest concentration of each compound that did not shift the neomycin dose-response curve (data not shown). At neomycin concentrations of 100, 200, and 400 μM either inhibitor demonstrated partial attenuation of Dihexa otoprotection, indicating at least partial reliance on their associated signaling proteins for protective effects (Figure 6A). Interestingly, when both inhibitors are co-administered the result is similar to each administered independently, suggesting that other signaling proteins may also contribute to protection (data not shown). Phosphorylation of Akt leads to activation of TOR, a molecular sensor of metabolism and cellular homeostasis (Wullschleger et al., 2006). We used the TOR inhibitor rapamycin to determine the extent to which activation of TOR is required in Dihexa-mediated protection. 10 μM rapamycin alone did not affect the neomycin dose-response curve (Figure 6B). However, co-treatment with 1 μM Dihexa and 10 μM rapamycin showed significant attenuation of hair cell protection at 50 and 100 μM neomycin. From these results we hypothesize that Dihexa-mediated protection relies, at least in part, on the activation of multiple downstream targets of the HGF/c-Met system (Akt-TOR and Ras-ERK).

### Modified Dihexa is not a hair cell protectant

**Figure 7:**
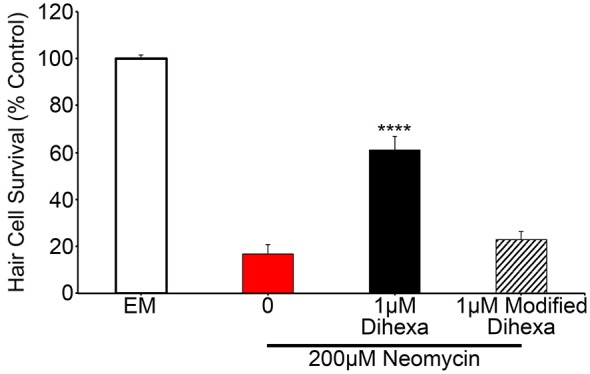
**A Dihexa variant that is missing an N-terminal amino group (modified) does not provide protection against 200 μM neomycin**. 1 μM Dihexa provides robust protection from 200 μM neomycin (*t*-test; *p* < 0.001) whereas 1 μM of modified Dihexa provides no significant protection (*p* = 0.34) when compared to 200 μM neomycin. Asterisks indicate significant difference from 200 μM neomycin control (****p < 0.001). *N* = 7 animals per treatment, error bars represent ± s.e.m.

To test the specificity of Dihexa for the observed protection, we synthesized an additional Dihexa variant that was missing an N-terminal amino group. We tested the modified Dihexa against 200 μM neomycin and observed no protection, suggesting that the current form of Dihexa has a high specificity for providing protection, likely via its specific interaction with native HGF (Figure 7).

### Dihexa does not protect hair cells from cisplatin

**Figure 8:**
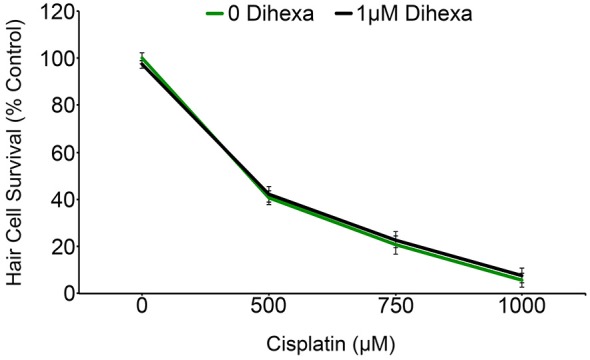
**Dihexa confers no protection from cisplatin ototoxicity**. Cisplatin dose-response curves with and without dihexa are nearly identical (Two-way ANOVA; Dihexa: *F*_(1,39)_ = 0.1845 *p* = 0.6699). *N* = 5–7 animals per treatment, error bars represent ± s.e.m.

Previous work with chemical and genetic cell death modulators suggests that cisplatin and aminoglycosides share some cell death pathways while also activating a distinct subset of signaling pathways (Owens et al., 2008; Vlasits et al., 2012; Coffin et al., 2013b). Animals exposed to Dihexa exhibit no shift in the cisplatin dose-response curve (Figure 8). This result signifies that Dihexa’s protection is likely by mediating the response of an aminoglycoside-specific target that is not shared by cisplatin.

## Discussion

We show that the HGF mimetic Dihexa protects lateral line hair cells from acute aminoglycoside ototoxicity. Previous work has reported that HGF/c-Met activation can confer hair cell protection from aminoglycoside damage, consistent with our results (Monahan and Samulski, 2000; Oshima et al., 2004; Kikkawa et al., 2009). However, the innate chemical properties of HGF, such as a short half-life, make it a poor therapeutic option (Appasamy et al., 1993). Additionally, HGF synthesis is prohibitively expensive, similar to other protein-derived neurotrophic factors (Swartz, 2001). These obstacles can be overcome by Dihexa, a synthetic, small molecule HGF mimetic that is orally bioavailable, metabolically stable, blood-brain barrier permeable, and inexpensive to synthesize (McCoy et al., 2013). Treatment with Dihexa has been shown to reliably activate c-Met activity through HGF-dependent interactions (Benoist et al., 2014). We think Dihexa represents an exciting new drug candidate for attenuating aminoglycoside ototoxicity.

Attenuation of aminoglycoside-induced hair cell death can result from two mechanistic categories: blockage of aminogly-coside uptake by hair cells or modulation of intracellular signaling pathways. Functional mechanotransduction is a known requirement for aminoglycoside entry into hair cells and its resulting ototoxicity (Alharazneh et al., 2011; Vu et al., 2013). Some compounds that protect hair cells from aminoglycoside exposure, for example tacrine and other quinoline ring-containing structures, do so by inhibiting aminoglycoside uptake (Ou et al., 2009, 2012). Even compounds with known intracellular targets, such as the estrogen receptor modulator raloxifene, may protect hair cells by inhibiting aminoglycoside entry, rather than acting via modulation of estrogen signaling (Vlasits et al., 2012). Here, we measured no difference in aminoglycoside uptake in hair cells treated with or without Dihexa, suggesting that Dihexa confers protection by modulation of cellular targets rather than blocking aminoglycoside uptake.

HGF/c-Met activates a diverse set of signal transducers capable of inhibiting cell death. For example, in various cell culture lines, c-Met phosphorylation can result in downstream activation of Akt, Erk/MAPK, JNK, or FAK signaling (reviewed by Organ and Tsao, 2011). We found that Dihexa-mediated protection requires activation of Akt, TOR, and MEK signaling, as inhibition of any of these targets attenuates Dihexa-mediated protection. Similarly, Insulin-like growth factor 1 hair cell protection from neomycin damage is lost by inhibition of Akt or MEK signaling, suggesting that growth factor otoprotection may operate on a similar set of downstream targets (Hayashi et al., 2013). Endogenous Akt activation also plays a survival role in cochlear hair cells in response to gentamicin ototoxicity (Chung et al., 2006). Activation of Akt or MEK in cell culture can prevent proteolytic cleavage of pro-caspase-9 thereby inhibiting classical caspase-dependent apoptosis (Cardone et al., 1998; Allan et al., 2003). The necessity of caspase activation for aminoglycoside-induced hair cell death is still debatable, so alternative, caspase-independent pathways may lie downstream of Dihexa-mediated protection (Cunningham et al., 2002; Matsui et al., 2003; Jiang et al., 2006; Coffin et al., 2013b). The pro-survival protein Bcl-2 regulates cell death by inhibiting pro-cell death members of the Bcl-2 family of proteins (Reviewed by Czabotar et al., 2014). Both HGF application and Akt activity result in increased Bcl-2 expression in cell culture (Pugazhenthi et al., 2000; Gordin et al., 2010). In human endothelial cells, HGF-induced cellular protection from hypoxia is dependent on Bcl-2 activity (Yamamoto et al., 2001). Bcl-2 overexpression protects hair cells from aminoglycoside exposure both *in vitro* and *in vivo* (Cunningham et al., 2004; Pfannenstiel et al., 2009; Coffin et al., 2013a). Given the complexity of signal modulators downstream of HGF/c-Met, additional work is needed to better understand what processes are important for Dihexa’s hair cell protection.

Understanding hair cell death mechanisms activated by specific ototoxins can help shed light on what molecular signals to therapeutically target for each toxic agent. Dihexa treatment protected hair cells from acute aminoglycoside treatment (neomycin or gentamicin) but conferred no protection from chronic gentamicin or cisplatin exposure. These data are consistent with known differences in cell death signals activated across these three groups (cisplatin, acute aminoglycosides, and chronic aminoglycosides). There are at least two processes of aminoglycoside-induced cell death that depend on both the particular aminoglycoside and exposure time (Coffin et al., 2009; Owens et al., 2009). Pharmacological inhibition of Bax, a Bcl-2 family member that promotes cell death, prevents neomycin but not gentamicin ototoxicity in zebrafish (Coffin et al., 2013a,b). In contrast, inhibition of p53 protects lateral line hair cells from acute neomycin or gentamicin as well as chronic gentamicin exposure (Coffin et al., 2013a). A previously identified hair cell protectant, PROTO1, showed robust protection from acute aminoglycoside exposure but only modest protection from the chronic process and offered no protection against cisplatin, similar to the result demonstrated here with Dihexa (Owens et al., 2008). Additionally, Vlasits et al. also identified multiple compounds from a FDA-approved drug screen with very similar protection profiles to Dihexa (Vlasits et al., 2012). These results describe a system of aminoglycoside-induced cell death that both shares molecular targets but also has unique features depending on aminoglycoside and exposure time. Dihexa’s protection from acute but not chronic aminoglycoside exposure further strengthens the two-phase model of aminoglycoside induced-hair cell death.

Future work will address the efficacy of Dihexa as a hair cell protect in mammalian models and against additional aminoglycosides. In summary, our study identifies a new hair cell protectant, Dihexa, that relies upon HGF/c-Met signal transduction for its protection. Due to its conscious design as an orally bioavailable compound, Dihexa is a strong clinical candidate as a hair cell protectant.

## Author contributions

*Participated in research design*: Phillip M. Uribe, Leen H. Kawas, Joseph W. Harding, Allison B. Coffin.

*Conducted experiments*: Phillip M. Uribe, Leen H. Kawas, Joseph W. Harding, Allison B. Coffin.

*Performed data analysis*: Phillip M. Uribe, Allison B. Coffin.

*Wrote or contributed to the writing of the manuscript*: Phillip M. Uribe, Leen H. Kawas, Joseph W. Harding, Allison B. Coffin.

## Conflict of interest statement

Joseph W. Harding is co-founder and shareholder of M3 Biotechnology, Inc. Leen H. Kawas is the CEO of M3 Biotechnology, Inc. M3 Biotechnology, Inc. is developing HGF mimetics and antagonists for the treatment of various disorders including dementia. Phillip M. Uribe and Allison B. Coffin hold no competing interests.
